# Demographic Profile and Clinical Characteristics of Adults with Down Syndrome in North-Eastern Romania

**DOI:** 10.3390/clinpract14050142

**Published:** 2024-08-30

**Authors:** Nicoleta Lefter, Irina Mihaela Abdulan, Alexandra Maștaleru, Maria-Magdalena Leon, Cristina Rusu

**Affiliations:** 1 Faculty of Medicine, “Grigore T. Popa” University of Medicine and Pharmacy, 700115 Iasi, Romania; nicoleta-n-perdun@d.umfiasi.ro; 2 Department of Medical Specialties I, “Grigore T. Popa” University of Medicine and Pharmacy, 700115 Iasi, Romania; alexandra.mastaleru@umfiasi.ro (A.M.); maria.leon@umfiasi.ro (M.-M.L.); 3 Clinical Rehabilitation Hospital, 700661 Iasi, Romania; 4 Department of Mother and Child Medicine, “Grigore T. Popa” University of Medicine and Pharmacy, 700115 Iasi, Romania; cristina.rusu@umfiasi.ro

**Keywords:** down syndrome, physical health, comorbidities, gender

## Abstract

(1) Background: Down syndrome is characterized by physical abnormalities, intellectual disability (ID), and specific patterns of other health issues. Additionally, individuals with DS are known to experience premature aging and early onset of certain age-related medical conditions. These conditions are linked to higher incident disability and reduced survival rates compared to the general population. (2) Methods: Between July 2022 and February 2024, we conducted a prospective, observational study in the Cardiovascular Rehabilitation Clinic at Iasi Clinical Rehabilitation Hospital. The study included 28 patients diagnosed with Down Syndrome and a control group. Interdisciplinary interventions were tailored to address the needs of a complex patient, incorporating cardiological, endocrinological, genetical, biological and developmental support. Data on physical health, cognitive development, and psychosocial well-being were collected. (3) Results: Our DS group consisted of 11 (39%) females and 17 (61%) males. Their age ranged from 20 to 55 years with a mean of 28.07 ± 9.51. All patients were unmarried, living in urban areas, without a partner but with family support. In the study sample, 96.4% of participants had three or more comorbidities. (4) Conclusions: The high prevalence of multimorbidity, combined with little medication, contributes to a high level of clinical complexity, which appears to be similar to the one of the older non-trisomic population. As individuals with Down syndrome transition into adulthood, they may require a more comprehensive and holistic approach to their healthcare.

## 1. Introduction

Down syndrome (DS) is the most prevalent human chromosomal disorder, occurring in approximately 1 in 792 live births in the US [1]. It is characterized by physical abnormalities, intellectual disability (ID), and specific patterns of other health issues. Individuals with DS also tend to experience premature aging and early onset of age-related medical conditions [2,3], leading to higher incident disability and reduced survival rates compared to the general population.

Over the past century, the life expectancy of individuals with DS has significantly increased. Newborns are now expected to live up to 60 years [4], a substantial improvement from the 1940s when their average life expectancy was only 12 years [5]. Thanks to medical advancements and improved services, individuals with DS can now enjoy life expectancies into their 60s [1].

As individuals with DS are now living longer, they are more likely to encounter age-related health issues earlier than the general population. Therefore, it is crucial to provide specialized healthcare for these adults who are at a higher risk for certain conditions such as dementia, early onset menopause, visual and hearing impairments, adult-onset seizure disorder, thyroid dysfunction, metabolic disorders like type 2 diabetes, and obesity.

With the changing demographics, DS should no longer be considered only a pediatric condition but rather a condition that requires attention throughout a person’s lifetime. However, managing patients with DS remains challenging due to limited evidence to guide care providers [6,7]. There is a lack of scientific literature describing the clinical characteristics of individuals with DS and addressing the screening for co-occurring medical conditions [8,9]. People with DS often experience multiple chronic illnesses, a condition known as multimorbidity, which is typically seen in older individuals [3]. Polypharmacy and multimorbidity are often interconnected, but there is currently insufficient data on medication use in adults with DS.

The EUROCAT data has been the subject of numerous studies concerning the prevalence of DS births in European countries [10,11]. However, the actual population size and prevalence of individuals living in European countries have only been approximately calculated. Between 2011 and 2015, it was estimated that there were 8031 live births of children with DS in Europe, with a live birth prevalence of 10.1 per 10,000 live births, and around 419,000 people with DS living in Europe [1]. More specific estimates of population size and prevalence were reported for a select few European countries, including England/Wales, the United Kingdom, Ireland, France, the Netherlands, and Denmark [12,13].

In Romania, there is no clear statistic or database that includes children with DS, let alone a clear record of adult patients. This makes it difficult to track them and provide appropriate health services. Including them in studies becomes even more difficult, as neither physicians have a database, nor do adolescents or adults with DS make regular visits to their specialist/family doctor except in case of a medical emergency. While urban patients have easier access to health services, rural patients come from disadvantaged families, most often with a low income, which further widens the gap between the person with DS and health services.

The research aims to enhance our knowledge of the health profile of adults with Down syndrome in North-Eastern Romania. It specifically examines the prevalence of chronic conditions and the use of medication among this population during their early adult years.

## 2. Materials and Methods

### 2.1. Study Design and Setting

Between July 2022 and February 2024, we conducted a prospective, observational study in the Cardiovascular Rehabilitation Clinic at Iasi Clinical Rehabilitation Hospital.

The study included 28 patients diagnosed with DS. It is important to note that the patients enrolled in this study were confirmed to have DS through genetic testing, specifically karyotyping, which identified the presence of trisomy 21, ensuring the reliability and accuracy of our findings.

Individuals were assessed through a standardized clinical protocol. Interdisciplinary interventions were tailored to address the needs of a complex patient, incorporating cardiological, endocrinological, genetical, biological and developmental support. Data on physical health, cognitive development, and psychosocial well-being were collected.

### 2.2. Study Participants

Out of 505 patients admitted during the specified period, 28 were included in our study. The inclusion criteria were age over 18, genetic testing that confirms the diagnosis, and signing of the informed consent form. The exclusion criteria were recent surgery, presence of neoplasia, presence of conditions associated with increased risk of falling, and refusal/failure to perform the proposed tests.

In order to compare the findings from the DS group, we also included a control group consisting of 29 patients from general population who were assessed outside of an acute episode of illness at their regular medical visit in the hospital.

### 2.3. Patients Evaluation

A single investigator performed the patient history and clinical examination to maintain objectivity. The collected demographic data covered age, gender, Body Mass Index, education level (grouped into no education, primary, secondary, and high school), and other diagnosed conditions. We also gathered details about family support and whether the patient lived in a single-parent household. The patient history included inquiries about medication, such as the types and frequency of medication, the person administering the medication, whether clinical reassessments supported the use of the medications, and the patient’s tolerance to them.

### 2.4. Ethical Approval

All included patients signed the informed consent form to be enrolled in the study. The study was approved by the Ethics Committee of both the Grigore T. Popa University of Medicine and Pharmacy Iași (certificate of approval dated 208/9 July 2022) and the Iași Clinical Rehabilitation Hospital (certificate of approval dated 13/22 June 2022).

### 2.5. Statistical Analysis

The statistical analysis was conducted using SPSS 20.0 (Statistical Package for the Social Sciences, Chicago, IL, USA). Categorical variables were presented as percentages and compared using the Chi-Square test. The normality of distribution for continuous variables was assessed using the Shapiro–Wilk test. Variables with a normal distribution were reported as mean values with standard deviations and compared using the Student’s *t*-test. Non-normally distributed continuous variables were reported as medians with interquartile ranges and compared using the Mann–Whitney U test. The threshold for statistical significance was set at *p* ≤ 0.05 for all analyses.

## 3. Results

Our research group consisted of 28 participants, with 11 (39%) being females and 17 (61%) being males. Their ages ranged from 20 to 55 years, with an average of 28.07 ± 9.51. In contrast, the control group had a higher average age of 47.41 ± 10.21. All individuals with DS were unmarried and lived in urban areas without a partner, but they had family support. In over 50% of cases, individuals with DS came from single-parent families. For more detailed demographic information on DS and the control group, please refer to Table 1.

Apart from age, there is a clear difference in educational status, with the control group being almost entirely high school or college graduates.

Regarding associated pathologies, there are significant differences between the groups (Table 2).

In the study groups, more than 90% of participants had three or more comorbidities. However, there was a difference in the associated pathologies. The DS group has predominantly ocular, hearing and thyroid diseases and behavioral issues (Table 2).

Upon examining the coexisting medical conditions by gender (Table 3), it was observed that both women and men experienced three or more health issues. Specifically, 72.72% of women had four or more conditions, while only 52.94% of men experienced the same number. 

We observed a significantly higher prevalence of myopia in women, with 100% of them having ophthalmologic impairments, compared to only 41.17% of men with documented vision issues. Furthermore, a statistically notable higher percentage of women experienced congenital heart disease and thyroid impairments. 

Regarding men, irritable bowel disease and hepatic steatosis were among the more common digestive disorders. In terms of the number of medications used for long-term treatment, it was found that the control group had a higher average, despite all patients having more than two coexisting medical conditions (Table 4).

In our research, the treatment for patients with DS included the following: 

- Zolpidem, a non-barbiturate hypnotic, was prescribed for insomnia in 1 out of 5 patients with a confirmed diagnosis. 

- Proton Pump Inhibitors were recommended for chronic gastritis in 7 out of 17 patients with a confirmed diagnosis. 

- Thyroid hormone replacement therapy was administered to 7 out of 24 patients with a confirmed diagnosis. 

- Analgesics/NSAIDs were used to manage neurological pathology such as lumbago/myalgia in 2 out of 11 patients with a confirmed diagnosis. 

- Antiplatelet agents were given for the prevention of thrombophilia in 1 patient with a confirmed diagnosis of CVI. 

- SSRI antidepressants/tricyclics were used for psychiatric treatment of behavioral disorders in 4 out of 8 patients with a confirmed diagnosis. 

- Anticonvulsants were prescribed for one patient with a confirmed diagnosis of epilepsy.

## 4. Discussion

Adults with Down syndrome often experience premature onset of comorbidities, which tend to be multifaceted. They exhibit early signs of senescence and are predisposed to developing various medical conditions at a younger age compared to the general population. They frequently present with cardiovascular complications, such as congenital heart anomalies, respiratory disorders (such as sleep apnea), and autoimmune disorders [14]. Moreover, they are at an increased risk of obesity, which can exacerbate their cardiac and vascular issues and elevate the probability of developing diabetes.

Thyroid dysfunction, particularly due to autoimmune thyroiditis, is a common comorbidity that requires lifelong management. Gastrointestinal issues, including celiac disease, are also prevalent. Moreover, individuals with DS are more susceptible to autoimmune disorders, complicating their health profiles further [12,15].

Overall, the early onset of comorbidities in adults with DS emphasizes the necessity for proactive, multidisciplinary medical care to meet their unique health needs and enhance their quality of life.

### 4.1. Obesity

Numerous studies demonstrate that being overweight and obese carries significant health hazards and is associated with increased rates of both mortality and morbidity. Furthermore, obesity presents a considerable challenge for adults with DS, leading to a range of health issues and negatively impacting their overall well-being. Various factors contribute to the elevated occurrence of obesity in this population, including metabolic, genetic, behavioral, and environmental factors.

In a cross-sectional study conducted at an outpatient clinic of a tertiary care hospital in Madrid, Spain, 51 adults with DS and 51 healthy controls were compared. The study found that adults with DS were significantly younger and there was a higher prevalence of men with overweight and obesity compared to the control group. After adjusting for age and gender, the analysis showed no differences in fasting insulin levels, homeostatic model assessment indexes, or lipid profiles between adults with DS and the control group [16].

The current study indicates a continued trend of male gender predisposition to obesity, with a slight increase among the control group, although it is not statistically significant. Interestingly, there were no instances of diabetes in the DS group, despite the expectation of a comparably overweight/obese population, unlike the control group where the percentage was 20.68%.

### 4.2. Myopia

The prevalence of myopia in the DS population in various studies ranges from 6.3% to 40.1% [17,18]. The largest study of a DS cohort in the United States showed a myopia prevalence of 22.5% [19], primarily influenced by genetic factors affecting eye development.

In a study by Angie Hong Chi Fong et al., it was found that there is a high prevalence of vision impairment among Chinese adults with DS. The main visually debilitating ophthalmological abnormalities were uncorrected refractive errors, high myopia, and cataracts. The study included 91 DS patients recruited through the Hong Kong Down Syndrome Association [20].

According to our studies, myopia is a common eye condition that seems to affect a higher number of female patients of all ages. It’s important to make proper vision care a priority for adults with DS. Regular eye examinations are crucial for early detection and correction, often through the use of glasses or contact lenses. Managing myopia effectively can enhance visual clarity, leading to improved daily functioning and overall quality of life. Additionally, other changes such as strabismus and astigmatism are also observed, but they occur in a smaller percentage, particularly within the DS group.

### 4.3. Hearing Impairment

Individuals with DS may require adjustments to hearing devices or increased monitoring of their hearing technology to manage their hearing loss effectively. These measures can support these individuals in optimizing their communication and cognitive abilities.

In our study, more than half of the patients had varying degrees of hearing loss, with no gender differences. This rate is consistently reported in studies [21]. When hearing impairment is also present, the physical challenges are worsened. Hearing loss can hinder communication and social interactions, making it more difficult for individuals to participate in group activities or follow instructions in physical settings. Additionally, intellectual disability was shown to make it particularly challenging to understand and respond to auditory cues or safety warnings during exercise.

### 4.4. Thyroid Disease

In the past 30 years, many publications have suggested a link between DS and thyroid disorders. They have shown altered levels of abnormal thyroxine (T4), triiodothyronine (T3), and/or thyroid-stimulating hormone (TSH). Most studies have reported a prevalence rate higher than that in the general population. An evaluation of these studies suggests a lifetime prevalence of approximately 25–30% [22]. The prevalence of hypothyroidism was found to be greater than that of hyperthyroidism, with hypothyroidism particularly becoming more common with aging [23]. Prasher et al. investigated thyroid dysfunction in 160 adults with DS (mean age 43.4 years; age range 17–76 years). They found that 35% had evidence of thyroid dysfunction, subclinical hypothyroidism 12%, definite hypothyroidism 8%, and hyperthyroidism 3% [24].

In the Intellectual Disability Supplement in The Irish Longitudinal Study on Aging (IDS-TILDA), thyroid disease was the most frequently reported health condition, affecting 37.4% (n = 55) of the participants. This condition was more common among females, with the highest prevalence observed in the 50–64 age group. However, only 2.7% (n = 4) of those affected sought endocrinological services [25]. Our study also found a high prevalence of thyroid issues among females. No patient in the control group had any thyroid disease.

### 4.5. Osteopenia/Osteoporosis

Osteoporosis and osteopenia have been extensively studied in the general population. However, they are relatively understudied among patients with DS [26]. In The Irish Longitudinal Study on Aging (IDS-TILDA), out of 147 participants with DS, 9.4% reported a doctor’s diagnosis of osteoporosis. This percentage is much lower than the prevalence of risk factors suggested [27]. Because there is no consistent approach to bone screening, an evident increased burden of risk factors for osteoporosis, and no clear guidelines for the risk burden among people with DS, further investigation is required.

None of the individuals with DS had a known history of the condition before participating in the study. These findings highlight the importance of conducting bone health screenings for this group of patients. In contrast, all individuals in the control group had previously undergone screenings, and none had been diagnosed with osteopenia/osteoporosis.

### 4.6. Heart Disease

DS is linked to several congenital defects and an increased risk of developing conditions later in life. For this reason, individuals with DS require comprehensive care and close monitoring. Congenital heart disease (CHD) affects 35–50% of patients with DS, and it is considered hemodynamically significant in two-thirds of cases [10]. Although survival rates have significantly improved after timely CHD repair, Körten et al. [28] reported a fourfold increase in mortality among DS patients compared to the general population. This higher mortality is likely due to DS-related comorbidities rather than the CHD itself. Previous studies in pediatric populations have shown that female cases are more prone to develop cardiac defects as compared to males [29]. Accordingly, our study revealed that the adult female population had a higher percentage of congenital heart defects compared to adult males.

It is interesting to note that none of the patients in the DS group had hypertension, whereas 72.41% of the patients in the control group were diagnosed and receiving treatment for it. Despite adults with DS having higher rates of certain traditional cardiovascular risk factors like dyslipidemia, obesity, and a sedentary lifestyle compared to the general population, they typically do not develop hypertension or experience significant cardiovascular events as they age.

The specific protective factors that prevent hypertension in T21 individuals are not fully understood. It is believed that genes like RCAN1 and DYRK1A, which are found on chromosome 21 and are more active in adults with DS, might have an important part in protecting against cardiovascular issues. These genes are believed to control the renin–angiotensin–aldosterone system (RAAS) and the production of neprilysin, which could help protect adults with DS from developing high blood pressure and might explain why they do not experience increased arterial stiffness [30].

In a research study involving 144 outpatient adults with DS, Real de Asúa et al. [12] found that none of the individuals had high blood pressure (HBP). This result was confirmed in other similar groups [31].

### 4.7. Gastrointestinal Issues

Adults with DS are prone to a range of gastrointestinal problems. Studies on these issues commonly mention conditions such as celiac disease, congenital structural changes, and gastroesophageal reflux [32]. Irritable bowel syndrome can be challenging to diagnose due to its non-specific clinical presentation and is often overlooked in screening studies. Our study found that irritable bowel syndrome was the most prevalent gastrointestinal condition among male patients.

Non-alcoholic fatty liver disease frequently coexists in individuals with Down syndrome, likely due to insufficient physical activity [33]. It is important to note that there is limited research on non-alcoholic fatty liver disease in adult patients with DS, as most studies have focused on the pediatric population. Despite the relatively small number of patients, our study uncovered a higher prevalence of this condition in adults with trisomy 21.

### 4.8. Multimorbidity and Medication

Most of the adults who took part in our survey had reliable access to primary health care, as most of them had a primary care doctor and reported getting regular medical check-ups. This relatively high level of health care access compared to others with developmental disabilities may be linked to a significant number of them living with their parents or other family members. Even though a large percentage of the patients had three or more concurrent medical conditions, they were taking a relatively low average number of medications, specifically 1.07 ± 1.21.

The co-occurrence of multiple diseases along with a low medication count, raises several concerns and can be interpreted in various ways depending on the context. It could indicate inadequate treatment, where the diseases are not being effectively managed due to limited access to health care or financial constraints, considering patient or caregiver preferences.

Alternatively, the approach to managing these conditions could be centered around non-pharmacological interventions such as lifestyle changes, diet, exercise, and other therapies. Another possibility is that the diseases are in their early stages, leading to a “wait and see” approach, whereby monitoring and lifestyle adjustments are prioritized before considering medications.

It is crucial for patients to have detailed discussions with their healthcare providers to ensure effective disease management, explore all available treatment options, and make adjustments to the treatment plan as necessary through regular monitoring and follow-up.

The small number of adults with DS participating in our study is due to several factors. Limited access to medical services is a result of poor healthcare infrastructure. There is also social stigma and discrimination, which result in isolation and hesitancy to participate in research. Furthermore, a lack of awareness about DS further limits participation. Geographical and socioeconomic barriers such as poverty and living in rural areas limit access to healthcare and research facilities. Many families require assistance with transportation, preventing them from reaching study sites. Additionally, contacting potential participants is challenging as many do not have registered phone numbers, and those who do often have outdated numbers. Ethical considerations in obtaining informed consent also contribute to cautious recruitment practices, resulting in smaller study groups.

## 5. Conclusions

The occurrence and clustering of multiple health conditions in individuals with DS follow distinct patterns compared to the general population. These differences have important implications for when and how healthcare screening, prevention, and treatment should be provided for people with Down syndrome.

The high prevalence of multimorbidity, combined with little medication, contrib-utes to a high level of clinical complexity, which appears to be similar to the one of the older non-trisomic population. As they grow into adulthood, people with DS may need a more comprehensive and holistic approach, commonly adopted in geriatric medicine.

Barriers to health care services and appropriate diagnosing and treatment options for adults with Down syndrome should be examined to increase addressability.

## Figures and Tables

**Table 1:** General characteristics of the study group.

	DS Group (n = 28)	Control Group (n = 29)	*p*-Value
Age, years (mean ± SD)	28.07 ± 9.51	47.41 ± 10.21	0.173
Gender			
- Women	11 (39.28%)	13 (44.8%)	0.705
- Men	17 (60.72%)	16 (55.2%)	
Education, *n* (%)			
- No Classes	3 (10.71%)	0 (0%)	-
- Primary	9 (32.14%)	0 (0%)	-
- Gymnasium	6 (21.42%)	1 (3.4%)	0.051
- High School	10 (35.71%)	18 (62.1%)	0.689
- University	0 (0%)	10 (34.5%)	-
Environment			
- Rural	0 (0%)	0 (0%)	-
- Urban	28 (100%)	29 (100%)	-
Family support	28 (100%)	29 (100%)	-
Single parent family	17 (61%)	0 (0%)	-

**Table 2:** Comorbidities in the study groups.

Comorbidities	DS Group (n = 28)	Control Group (n = 29)	*p*-Value
Number of diseases			
- 0	1 (3.6%)	0 (0%)	-
- 1	0 (0%)	0 (0%)	-
- 2	0 (0%)	0 (0%)	-
- 3	9 (32.1%)	11 (37.9%)	0.789
- 4 or more	18 (64.3%)	18 (62.1%)	0.875
Visual impairment			
- strabismus	3 (10.71%)	0 (0%)	-
- myopia	10 (35.71%)	0 (0%)	-
- astigmatism	5 (17.85%)	0 (0%)	-
Thyroid disease	16 (57.1%)	0 (0%)	-
Hearing impairment	15 (53.57%)	0 (0%)	-
Congenital heart disease	9 (32.2%)	2 (6.9%)	0.197
Hypertension	0 (0%)	21(72.41%)	-
Obesity	11 (39.28%)	12 (41.4%)	0.874
Diabetes mellitus	0 (0%)	6 (20.68%)	-
Hypercholesterolemia	10 (35.7%)	20 (69%)	0.010
Hypertriglyceridemia	3 (10.7%)	16 (55.2%)	0.0002
Osteopenia	16 (57.1%)	0 (0%)	-
Osteoporosis	6 (21.4%)	0 (%)	-
Insomnia	5 (17.85%)	4 (13.8%)	0.680
Gastrointestinal disease			
- Chronic gastritis	17 (60.7%)	12 (41.4%)	0.149
- Biliary lithiasis	3 (10.7%)	1 (3.4%)	0.291
- Irritable bowel disease	11 (39.3%)	6 (20.7%)	0.741
Liver steatosis	16 (57.1%)	14 (48.27%)	0.430
Epilepsy	1 (3.6%)	0 (0%)	-
Disk hernia	3 (10.71%)	2 (6.9%)	0.618
Lumbago	11 (39.28%)	2 (6.9%)	0.003
Behavioral Issues	10 (35.71%)	0 (0%)	-
Chronic venous insufficiency	12 (42.85%)	8 (27.6%)	0.234

Note: values are reported as mean ± SD or frequency (%).

**Table 3:** Gender-related comorbidities in DS group.

Comorbidities	Female (n = 11)	Male (n = 17)	*p*-Value
Number of diseases			
- 0	0 (0%)	2 (11.76%)	-
- 1	0 (0%)	1 (5.88%)	-
- 2	0 (0%)	1 (5.88%)	-
- 3	3 (27.27%)	7 (41.17%)	0.47
- 4 or more	8 (72.72%)	9 (52.94%)	0.31
Visual Impairment			
- strabismus	1 (9.09%)	2 (11.76%)	0.83
- myopia	7 (63.63%)	3 (17.64%)	0.01
- astigmatism	3 (27.27%)	2 (11.76%)	0.31
Thyroid Disease	9 (81.81%)	7 (41.17%)	0.03
Hearing Impairment	6 (54.54%)	9 (52.94%)	0.93
Congenital Heart Disease	8 (72.72%)	1 (5.88%)	0.000035
Obesity	4 (36.36%)	7 (41.17%)	0.80
Hypercholesterolemia	4 (36.36%)	6 (35.29%)	0.95
Hypertriglyceridemia	1 (9.09%)	2 (11.76%)	0.83
Osteopenia	5 (45.45%)	11 (64.70%)	0.33
Osteoporosis	2 (18.18%)	4 (23.52%)	0.74
Insomnia	1 (9.09%)	4 (23.52%)	0.34
Gastrointestinal disease			
- Chronic gastritis	6 (54.54%)	11 (64.70%)	0.60
- Biliary lithiasis	2 (18.18%)	2 (11.76%)	0.32
- Irritable bowel disease	3 (27.27%)	9 (52.94%)	0.13
Epilepsy	0 (0%)	1 (5.88%)	-
Disk hernia	3 (27.27%)	0 (0%)	-
Lumbago	4 (36.36%)	7 (41.17%)	0.80
Behavioral Issues	2 (18.18%)	8 (47.05%)	0.12
Chronic venous insufficiency	4 (36.36%)	8 (47.05%)	0.29
Liver steatosis	2 (18.18%)	8 (47.05%)	0.12

**Table 4:** Multimorbidity and medication use.

	Total Group (n = 28)	Control Group (n = 29)	*p*-Value
Number of medications, (mean ± SD)	1.07 ± 1.21	3.93 ± 2.81	<0.00001
Multimorbidity (>2 chronic diseases)	27 (96.42%)	29 (100%)	0.973

## Data Availability

The data that support the findings of this study are available from the corresponding author upon reasonable request.
